# Implementation of a post-arrest care team: understanding the nuances of a team-based intervention

**DOI:** 10.1186/s13012-016-0463-x

**Published:** 2016-08-04

**Authors:** Katie N. Dainty, Elizabeth Racz, Laurie J. Morrison, Steven C. Brooks

**Affiliations:** 1 Rescu, Li Ka Shing Knowledge Institute, St. Michael’s Hospital, 30 Bond Street, Toronto, ON M5B 1W8 Canada; 2 Department of Emergency Medicine, Queen’s University, c/o 76 Stuart Street, Kingston, ON K7L 2V7 Canada

**Keywords:** Cardiac arrest, Multidisciplinary care teams, Resuscitation, Implementation science, Qualitative methods

## Abstract

**Background:**

Despite advances in the management of sudden cardiac arrest, mortality for patients admitted to hospital is still greater than 50 %. Lack of familiarity and experience with post-cardiac arrest patients and lack of interdisciplinary collaboration between emergency and ICU staff have been highlighted as potential barriers to optimal care. To address these barriers, a specialized Post Arrest Consult Team (PACT) was implemented at two urban academic centers.

Our objective was to describe the PACT implementation from the participant perspective in order to explore potentially mitigating factors on effectiveness of the intervention and inform other institutions who may be considering a similar approach.

**Methods:**

Using an ethnographic style approach, we collected data throughout the implementation period using both key informant interviews and non-participant observation. The data were analyzed using interpretive descriptive analysis techniques.

**Results:**

The PACT intervention was taken up differently in each of the two participating institutions. Participants spoke about the difficulty in maintaining a dynamic interaction between the team members and a shared sense of purpose, the challenge of off-service consulting and the impact of the lack of data feedback to support whether the project was effecting change.

**Conclusions:**

It appears that purposefully creating a “sense of team,” the team composition and organizational culture and provision of performance feedback are important facilitators to ensuring uptake of a team-based intervention like the PACT model. Reporting of the intervention design and actual implementation experience like we have done here is crucial to allow readers to judge the quality of the study, to properly replicate it, and to contemplate how various factors may influence the outcome of a complex intervention.

## Background

Out-of-hospital cardiac arrest (OHCA) is a common and lethal public health problem. The North American incidence of emergency medical services (EMS)-treated cardiac arrest is estimated to be 52.1 per 100,000 people per year [1]. Despite advances in the immediate management of sudden cardiac arrest over the past six decades, mortality for patients admitted to hospital is still greater than 50 % [1].

Several international position statements have defined best practices for patients with post-cardiac arrest syndrome, including the rapid induction of targeted temperature management, selective use of percutaneous coronary intervention, assessment for an implantable cardioverter-defibrillator, and appropriately delayed neuroprognostication [1, 2]. Numerous potential barriers to the delivery of optimal post-cardiac arrest care have also been identified in the literature [3–5]. For example, lack of familiarity and experience with post-cardiac arrest patients due to relatively low annual volumes of this type of patient at any given institution has been consistently cited [5]. A lack of interdisciplinary collaboration between emergency department (ED) and intensive care unit (ICU) staff and lack of access to advanced technology and specialized human resources are commonly identified as organizational barriers.

Previous research has demonstrated improved survival with the implementation of standardized bundled care plans for the post-cardiac arrest patient [6–8]. Improved outcomes have also been demonstrated for patients suffering from other types of complex, acute illnesses such as severe trauma and ST-elevation myocardial infarction with the implementation of specialized interdisciplinary teams and evidence-based systems of care [2, 9–13]. With this in mind, the intervention was designed to directly address barriers to optimal care for post-cardiac arrest patients by implementing a specialized Post Arrest Consult Team (PACT) at two urban academic centers. The primary analysis of this intervention, which is published elsewhere [14], involved a quantitative comparison of process and clinical outcomes with concurrent and historical controls from several other hospitals that did not have PACT implementation within the same geographical region.

This paper represents the results of an integrated qualitative evaluation designed to study the PACT implementation process in detail and identify potential mediating factors perceived by frontline clinical staff in the PACT institutions. To date, there is little research on how health organizations take up, support, and embed complex innovations [15], such as the PACT, to inform implementation of other similar interventions. Research on the impact of quality implementation of programs and services has shown that without a focus on implementation best practices, outcomes may not be achieved as expected, and in some cases a poorly implemented program may produce harmful results [16]. As the intervention study was being planned, it was recognized that understanding this piece of the project would be essential to comprehensively evaluate the effectiveness of this novel approach and expand our understanding of the quantitative study outcomes.

### The PACT intervention (as designed)

The focus of the intervention was to provide an institution-wide, standardization of post-cardiac arrest care and collaboration across clinical specialties by creating a new "post-arrest consult team” —the PACT. The PACT was designed to improve the consistency of care delivery through a standardized approach, collaboration between the consultation team and the primary services caring for the patient and education to ensure that all patients receive optimal care based on best evidence and current guidelines. Evidence-based clinical pathways, which prescribed evaluation and management strategy guidance to the PACT, were developed through a consensus by a group of investigators comprised of a physician and nursing specialists in emergency medicine, cardiology, and intensive care. These clinical pathways were derived from the 2010 American Heart Association Emergency Cardiovascular Care and Cardiopulmonary Resuscitation Guidelines [17].

The PACT was operationalized through an on-call team including a physician and nurse available for consultation 24 h a day. The PACT physician was asked to be available for urgent (within 30 min) bedside consultations during business hours (9 am to 5 pm, Monday to Friday) and available for phone consultations during off hours (after 5 pm, weekends and holidays) with the option to come to the hospital for bedside assessment when required. The team’s main objective was to provide timely expertise and collaborative hands-on assistance to the treating physicians and nurses who maintained primary responsibility for the patient while they were in hospital.

The immediate emphasis of PACT involvement was on ensuring the rapid induction of targeted temperature management, avoidance of hyperoxia and hypocarbia, and assessment for urgent coronary angiography. These best practices were to be addressed during initial consultation by the PACT, usually in the ED. Ongoing follow-up by PACT with the patient on a daily basis was planned for the first 72 to 96 h after arrest so that the electrophysiology and delayed neuroprognostication pathways could be addressed and supported. During these consultations, PACT members were encouraged to leave a standardized note in the patient chart and discuss items of recommendation directly with the primary service as necessary.

Similar to other consult teams in the hospital, PACT members were to carry pagers when they were on-call. The team could be activated via one of two mechanisms: (a) by the ED team through the hospital switchboard in a fashion similar to activating a CODE BLUE cardiac arrest response or (b) via an automatic email alert facilitated by the local emergency medical services when a post-cardiac arrest patient was delivered to one of the PACT hospitals. When the email alert was received, the PACT was to call down to the ED and confirm that the patient had arrived alive. If the patient was alive, the PACT was to head down to the ED to assist with post-resuscitation care.

**Fig. 1:**
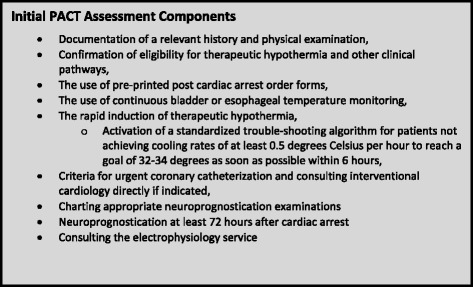
Initial PACT assessment components

The components of the initial PACT assessment are outlined in Fig. 1. Upon receiving a request for consultation, on-call PACT members were asked to assess the patient as soon as possible. If the physician was out-of-hospital, the PACT nurse completed the initial bedside assessment, with physician consultation by phone. In a similar fashion to many other clinical consult services available in most hospitals, the PACT worked in a collaborative way with the most responsible care providers in both the EDs and ICUs. When the PACT was called to assist with a patient, the requesting physician remained in the role of the most responsible physician (MRP). The MRP is the emergency physician assigned to the patient or the physician under whose care the patient has been admitted to hospital. The MRP continued to direct components of ongoing resuscitation and general critical care, and the PACT provided support, expert guidance, and “hands-on” human resources during the management of these complex patients.

Subsequent bedside assessments were made on a regular 24-h basis and additionally as required by the clinical scenario. Each visit included a review of the PACT clinical pathways, a note in the patient chart by the PACT clinician, and verbal communication with assigned clinical team members.

The primary initial task for the PACT nurse was to facilitate the rapid induction of targeted temperature management for eligible patients by providing guidance and assistance to the primary nurses. The PACT nurse would then collaborate with the PACT physician on all other aspects of post-arrest care. This nursing role was designed to be similar to other specialized nursing consultants such as the clinical nurse specialist component of many stroke or trauma teams. The PACT nurse also screened daily hospital admissions for missed cardiac arrests, which may be appropriate for follow-up from the PACT.

PACT physicians and nurses were also intended to provide interprofessional education to the ICU, ED, and cardiac care unit (CCU) personnel through presentations at educational rounds and orientation sessions, as well as informal knowledge translation at the bedside.

The principal investigator and study team intended to facilitate ongoing education and quality assurance by using data on care processes and clinical outcomes as a feedback tool. There were to be monthly rounds scheduled for all PACT and steering committee members in order to exchange feedback on team operations. Reviews of PACT cases were employed to fine-tune the activities of the team for maximal impact.

## Methods

### Study setting

The PACT project was implemented in two academic health science centers in Toronto, Ontario, and qualitative data collection for this study occurred specifically in the ED and ICU at both centers. Hospital A was a level 1 trauma center with over 1200 beds and 40,000 emergency room visits per year. Hospital B was also an urban level 1 trauma and critical care center with close to 500 beds and 75,000 ED visits annually. On average, local ambulance services transport approximately 60 out-of-hospital cardiac arrests to each institution annually.

### Study design

Using an ethnographic approach, the data were collected throughout the implementation period using both key informant interviews and non-participant observation. The interviews followed a semi-structured format using an interview guide developed by the steering committee and informed by the objectives of the larger qualitative study. This format allowed the interviewee to guide the conversation while at the same time providing some direction around certain topics. Interviews lasted approximately 20–30 min and were conducted in person by a PhD trained qualitative researcher (KND). All interviews were digitally recorded and transcribed verbatim by an external transcription service.

A purposive sampling strategy [18] was used to select the participants for interviewing due to the specific type of participants required, i.e., those who were part of the PACT or interacted with the PACT during the intervention period. All physicians, nurses, and health discipline professionals (respiratory therapists and occupational therapists) from the ICU and ED from both sites who are involved with the PACT were asked to participate via email and at study team meetings (*n* = 36). All of the participants agreed to be interviewed. Sampling continued until the investigators felt that thematic and participant saturation had been reached [19].

Qualitative observation during and after implementation was done to see first-hand how the intervention was implemented compared to how it was originally designed, how staff from the various units interacted with each other, and what specific attributes of the implementation appeared to be influential in the uptake of the PACT within the participating organizations. The qualitative researcher (KND) carried a pager and was notified of each cardiac arrest in which the PACT was activated. When possible, she met the incoming ambulance in the ED and observed the entire care process and team interaction through to ICU admission (approximately 1 h per case). Observations were also conducted at the 24-h follow-up point in the ICU and at all study team meetings. All clinical team members and PACT members were made aware of the ongoing observational data collection and given the option to decline to be observed. If a team member did not wish to be observed, the researcher would cease observation of that case and return on a different day. No clinical team members or PACT members declined participation at any point in the study.

Interview and observational data were analyzed using an interpretive descriptive approach [20]. All analysis was done independently by the lead researcher and compared with research colleagues at a later date. Descriptive codes were first attached to segments of the text in each transcript. The descriptive codes were then grouped in to broad topic-oriented categories, and all text segments belonging to the same category were compared. Ultimately, patterns and themes within the data were interpreted into key learnings through an inductive, iterative process, in order to make sense of the most important ideas to be conveyed and reflect on their meaning in a new manner [20].

## Results

Data were collected and analyzed over the course of the 2-year implementation. During this time, 20 key informant interviews were conducted at several time points and over 40 h of observation between the two sites. The qualitative data collected over the course of the implementation highlighted three major themes which represent key factors from the participant perspective that are highly informative for future implementations of PACT type interventions. In no particular order, they were: (a) the importance of creating a sense of team, (b) the influence of team composition, and (c) the importance of ongoing performance feedback.

### The importance of creating a “sense of team”

While PACT RNs and MDs were generally trained together in terms of the clinical knowledge required to function in the role and required data collection, no explicit team building was done as part of the implementation plan. In some cases, the nurse-physician teams had not worked together before, and initial communication was difficult in these situations. At each site, there were several clinicians involved as PAC Team members; however, they all took turns “on call” on a rotating basis and therefore never actually worked together. Due to the high variability in the frequency of the actual calls, some clinicians consulted on less than five cases over the 2 years of the study. This made it very difficult to maintain a dynamic interaction between the team members and a shared sense of purpose.

It wasn’t a natural constituency for the team but we didn’t really do anything to ‘pull’ the team together in a more formal way, for instance focusing on team building exercises, etc. The fact that the team would work together was a little bit taken for granted in an environment of balkanized care. (Physician PACT member 3)

The “team” concept is very seductive but then you have a team for everything and the responsibilities and roles are confusing. (ICU nurse manager 2)

### Team composition

A key feature of the PACT intervention was its intended ability to respond immediately to an incoming cardiac arrest ensuring timely initiation of best practices and then to follow the patient through the course of their stay in hospital. Due to different resource and organizational issues at the study site hospitals, the availability of different nursing and physician resources for recruitment to the PACT varied. As described above, in hospital A, a small core of two to three daytime specialist nurses without primary patient assignments (e.g., clinical resource nurses) took the role of PACT RN during regular business hours. These were senior nurses who had been at the hospital for a significant amount of time and were well known within the organization. For off hours, PACT duties were incorporated into the responsibilities of a pre-existing 24-h critical care rapid response team (CCRT), an on-call team consisting of ICU physicians and nurses who provide 24/7 emergency response for admitted patients who experience a medical emergency or acute worsening of their condition (more universally known as medical emergency teams (METs)).

In hospital B, PACT duties were assigned daily to a nurse working in the cardiovascular ICU. There were approximately 15–20 CVICU nurses on the PACT roster. At least one was identified as being on-call at all times unless none were working in the hospital. These nurses had primary patient care assignments in the CVICU while they were on-call for PACT. Because their primary responsibility was to their CVICU patients, availability for immediate bedside consultation was variable. They were asked to respond to the PACT call as soon as they could safely arrange coverage of their CVICU responsibilities. Traditionally, CVICU nursing staff do not practice outside of the CVICU, and therefore, PACT represented a departure from their usual work environment.

As bedside nurses we are “doers” so the consulting role was very different for us; plus we don’t traditionally go to the ED so we don’t know the system there and it’s a bit hard to parachute in. (PACT nurse 1)

ED does need help to get started and ensure they follow all the protocols but it really should be one of their own to help them; someone that understands how they work. (PACT nurse 4)

It was noted through the observational data collection that the placement of the team with an existing CCRT/MET consult service seemed to allow for more flexibility in responding to calls; however, both teams found the consultation role in the ED challenging. The ED and ICU are both very complex clinical units with their own cultures, systems, standard operating procedures, and command hierarchies, and so the assumptions that clinicians (nurses and physicians) would automatically be able to crossover and assimilate from ICU to the ED environment were noticeably flawed. It seemed that very little clinical knowledge regarding the best practices was translated at the initial bedside consult and much of the time appeared to be spent observing and identifying the right time to initiate conversation within the chaotic environment. In many cases, the patient had already been transported up to the ICU by the time the PACT member arrived in the ED, and so the opportunity to encourage early initiation of the evidence-based guidelines was often diluted. There was more discussion at the 24-h follow-up visit, particularly between the PACT physician and the attending physician now responsible for the patient. However, this often appeared awkward as the PACT could only provide advice and the care orders ultimately were written by the non-PACT physician.

### The importance of performance feedback

Audit and feedback can be defined as a summary of clinical performance (audit) over a specific period of time and the provision of that summary (feedback) to individual practitioners, teams, or healthcare organizations [21]. Regular feedback of outcome data were an intended activity of the PACT intervention. Unfortunately, due to unforeseen database issues, the research team was unable to provide specific PACT feedback reports to the teams until the last few months of the study.

While normally this may not have been an issue, health care has become heavily reliant on the reporting of data as the measure of success and failure. When interviewed, many of the participating clinicians felt that the PACT was “a good idea” but in the end struggled with whether or not the PACT approach was effective due to the lack of data. There also was not a timely debrief of each case, and so responsive changes were not possible. Given that the intervention is rather intensive in terms of time and resources, the lack of data resulted in a loss of engagement on the part of the teams as they began to question the capacity of the study to effect change.

Our core nursing leadership did the lion’s share of the work on good will because the Physician enthusiasm was low and waned significantly with the lack of feedback on whether we were having any effect. (PACT nurse 2)

There were cooling reports provided but they didn’t seem to include all of the patients we were seeing? It didn’t seem to match the numbers [of patients] we thought we were seeing and so the data wasn’t that useful. (PACT nurse 6)

Strong site champion engagement in team debriefs, review of overall institutional post-arrest reports and report back to the teams in both the EDs and ICUs also appeared to be lacking. The idea of the intervention was to improve the overall institutional approach to post-arrest care, and this seemed to be lost during implementation. Arrival of a surviving out-of-hospital cardiac arrest patient is a relatively rare event within any hospital, and therefore, it is difficult to get a sense of the impact of an intervention in day-to-day practice, and therefore, performance feedback can be a way to maintain attention on the program. Kluger and DeNisi’s [22] Feedback Intervention Theory, a framework from industrial/organizational psychology, proposes that feedback interventions generally work by providing new information that redirect recipients’ attention toward (or in some cases away from) the task; phenomena that redirect attention toward the details of the task tend to strengthen feedback’s effect on task performance. The lack of feedback about the impact of PACT on processes or clinical outcomes may have actually worked to weaken the team member’s attention to the intervention, especially in light of increasing competing interests for the attention of clinicians. Without data, there was also no way for the team to know what parts of the PACT intervention were effective and which parts may need modification, i.e., supporting a continuous quality improvement approach to the intervention.

## Discussion

The objective of this paper was to examine the potential impact that implementation choices may have had on the effectiveness of the PACT model from the point of view of participating team members. While purposefully designed as a multi-method study from the outset, largely negative quantitative study results have turned greater attention to understanding what contextual factors may have influenced the results of the intervention. The quantitative concurrent control study was able to demonstrate that a specialized post-cardiac arrest consult team was able to impact the occurrence of premature withdrawal of life-sustaining therapy but did not improve rates of targeted temperature management, the use of coronary angiography, or electrophysiology assessments prior to hospital discharge [14]. The analysis and observations reported here reveal three key findings that may have had a fundamental impact on the PACT’s ability to function as intended and to influence the uptake of most of the evidence-based guidelines.

Firstly, participants strongly conveyed the importance of the need to actively *create* a sense of team in a project like this. While referred to as a “team” and requiring members to work on related task, the members of PACT did not necessarily work with one another dynamically and have a shared past, a shared goal, or a common fate, and so, it may have been helpful to place more emphasis on recognized teambuilding strategies for this type of intervention. A systematic concept analysis in 2008 concluded that teamwork in healthcare is “a dynamic process involving two or more healthcare professionals with complementary backgrounds and skills, sharing common health goals and exercising concerted physical and mental effort in assessing, planning, or evaluating patient care” [23]. To work effectively together, team members must possess specific knowledge, skills, and attitudes, such as the skill in monitoring each other’s performance, knowledge of their own, and teammate’s task responsibilities, and a positive disposition toward working in a team [24]. The Healthcare World has become so specialized that our care of complex patients such as cardiac arrest victims is dependent on collaborative teamwork and is by definition interdependent; however, we cannot fall prey to assumptions that people who do related tasks are a team. Training and organization of care within a hospital setting (subspecialty training, physician vs. nurse training, program-based organizational structures, etc.) tend to support patient ownership and silos of care that start and end at the doors of each unit. The intended advantages of interventions like PACT, which specifically leverage the creation of interprofessional and cross-disciplinary teams, can only be realized if an effort is made to build those specific behaviors recognized as critical to good *team* performance [25].

Secondly, within the field of medicine, the role of a specialist consultant is a unique one. Typically a patient is assigned to a lead attending physician and nurse(s)/nursing team, and a “consult” from another service is only requested when the attending physician would like the expert opinion of a colleague trained in a specific area of medicine, such as cardiology and neurology. The consult is “sought” by the attending creating a “pull” form of knowledge transfer [26]. The PACT also departed from the usual practice of more specialized teams arriving for consult and often “taking over” the care of the complex patient, such as in the case of stroke or trauma teams. Having a team arrive unrequested to help and consult on care but not assume responsibility is a very different model—more of a “push” form of knowledge translation [26], and the staff engagement with such an approach, for both a new consultant and the consultee, is something which must be monitored and fostered based on our experience. This departure from normal consultation practices may have caused some role confusion among the PACT members and also the healthcare providers who are part of the unit that is currently managing the patient (either the ED or ICU).

Finally, the significance of providing performance feedback to the teams throughout the intervention (not just at the end of the study) was discussed almost unanimously by participants. Audit and feedback is a widely recognized strategy to improve professional practice either on its own or as a component of multifaceted quality improvement interventions. Designing a program to enable practice improvement requires departments to have a good understanding of what drives people to behave in the way that they do. This is based on the belief that healthcare professionals are prompted to modify their practice or take up a new practice when given performance feedback showing that their practice is inconsistent with a desirable target [21]. Clinicians are conditioned to looking to research data as evidence of effect, and so a lack of data to support the contribution of the PACT intervention to improved patient outcomes had a demoralizing effect on the PACT members and units. With resource-intensive interventions such as this one, it is important for team focus and morale that they have a way to know they are making a difference to patient outcomes and if they are not, how the process could be tweaked in situ to ensure the impact of the participant’s efforts is felt.

Providing a more indepth report of the actual implementation of an intervention like this is crucial to allow readers to judge the quality of the primary study and contemplate how various implementation factors may have influenced the reported outcomes [27]. The findings indicated that while it may appear simple enough in design, the introduction of a new specialized care team like PACT requires very close attention be paid at the design phase to what are largely sociologic realities of interprofessional and organizational culture in order to ensure that the intervention has the best possible chance of effecting the desired change. This speaks to what is commonly referred to in implementation science as a lack of intervention fidelity. Intervention fidelity refers to the extent to which the intervention was delivered as it was intended and more importantly in the case where it differed, why that occurred, and what it meant for the study outcomes [27]. The PACT intervention was implemented differently than originally intended due to both organizational factors and a lack attention to and supports for the complexities of a team-based intervention. It is actually possible that in fact this intervention did not achieve the desired effect largely because the advantage of using a response team to improve the use of evidence-based practices may have been diluted.

The findings reported here are based on interviews and observations in only two organizations; however, the data were collected in a rigorous, longitudinal, and representative way, and so it was felt that the results presented here are transferable and useful for organizations looking to implement similar process improvement interventions. The methodology for this work purposefully places interviews, rather than participant observation, at the center of the research design. This allowed for the use of the participants’ report of their experience of the intervention, particularly multidisciplinary stories of the PACT “work,” to investigate some of the less obvious implementation influences.

## Conclusions

A critical element often missing from published quantitative reports of quality improvement interventions is a discussion of intervention fidelity, implementation realities, and their impact on engagement with a new practice and the evaluation of its effectiveness. By outlining how the intervention was actually implemented and how that differed from the intended design and the nuances that may be unique to team-based interventions, we hope to have drawn attention to some of the often overlooked complexities of such seemingly simple changes in interdisciplinary practice. This research provides insight into the impact of purposefully creating a “sense of team,” team composition, and consult culture and ongoing provision and engagement with performance feedback as important facilitators to ensuring effective uptake and impact of team-based interventions like the PACT model.
